# Universal multilayer network embedding reveals a causal link between GABA neurotransmitter and cancer

**DOI:** 10.1186/s12859-025-06158-5

**Published:** 2025-06-02

**Authors:** Léo Pio-Lopez, Michael Levin

**Affiliations:** 1 Allen Discovery Center, Tufts University, Medford, USA; 2 Wyss Institute for Biologically Inspired Engineering, Boston, 02115 USA

**Keywords:** Network embedding, Multi-layer network, Drug repositioning, GABA, cancer, AI, Machine learning, Drug discovery

## Abstract

**Background:**

The volume and complexity of biological data have significantly increased in recent years, often represented as network models continue to increase at a rapid pace. However, drug discovery in the context of complex phenotypes are hampered by the difficulties inherent in producing machine learning algorithms that can integrate molecular-genetic, biochemical, physiological, and other diverse datasets. Recent developments have expanded network analysis techniques, such as network embedding, to effectively explore multilayer network structures. Multilayer networks, which incorporate various nodes and connections in formats such as multiplex, heterogeneous, and bipartite networks, provide an effective framework for merging diverse and multi-scale biological data sources. However, current network embedding methods face challenges and limitations in addressing the heterogeneity and diversity of these networks. Therefore, there is an essential need for the development of new network embedding methods to manage the complexity and diversity of multi-omics biological information effectively.

**Results:**

Here, we report a universal multilayer network embedding method MultiXVERSE, which is to the best of our knowledge the first one capable of handling any kind of multilayer network. We applied it to a molecular-drug-disease multiplex-heterogeneous network. Our model made new predictions about a link between GABA and cancer that we verified experimentally in the Xenopus laevis model.

**Conclusions:**

The development of MultiXVERSE represents a significant advancement in the integration and analysis of multilayer networks for biological research. By providing a universal, scalable framework for multilayer network embedding, MultiXVERSE enables the systematic exploration of molecular and phenotypic interactions across diverse biological contexts. Our experimental validation of the predicted link between GABA and cancer using Xenopus laevis underscores its capability to generate biologically meaningful hypotheses and accelerate breakthroughs in multi-omics research. Future directions include applying MultiXVERSE to additional multi-omics datasets and integrating it with high-throughput experimental pipelines for systematic hypothesis generation and validation, particularly in drug discovery. Beyond its biological applications, MultiXVERSE is a versatile tool that can be utilized for analyzing multilayer networks in a wide range of fields, including social sciences and other complex systems. By offering a universal framework, MultiXVERSE paves the way for novel insights and interdisciplinary collaborations in multilayer network research.

## Introduction

Network graph models are highly effective for depicting real-world objects through their relationships and interactions [1]. They offer valuable insights into the connections between different entities and are utilized as tools to investigate complex systems across various fields [2–4]. A significant challenge in machine learning involves converting high-dimensional graph-based data into a feature vector. Indeed, these methods were originally designed for vector data and cannot be directly applied to biological datasets such as biological networks [5, 6]. Network embedding, also known as graph representation learning, addresses this issue by transforming network data into formats compatible with conventional machine learning tools, thereby broadening the scope of machine learning applications in network analysis.

Network embedding techniques have proven highly effective across numerous applications, including community detection, node classification, and link prediction. Capable of handling vast networks with millions of nodes, these techniques are particularly valuable in the era of big data. Consequently, network embeddings are increasingly used to analyze various large-scale networks, such as social [3], neuronal [4] or molecular networks [2, 7, 8].

The volume and complexity of biological data have significantly increased in recent years, often represented as multilayer network models [1, 9]. Multilayer networks, which incorporate various nodes and connections in formats such as multiplex, heterogeneous, and bipartite networks, provide an especially effective framework for merging diverse and multi-scale biological data sources [1, 9]. However, current network embedding methods face challenges and limitations in addressing the heterogeneity and diversity of these networks [8]. Therefore, there is an essential need for the development of new network embedding methods to manage the complexity and diversity of multilayer networks effectively.

**Fig. 1:**
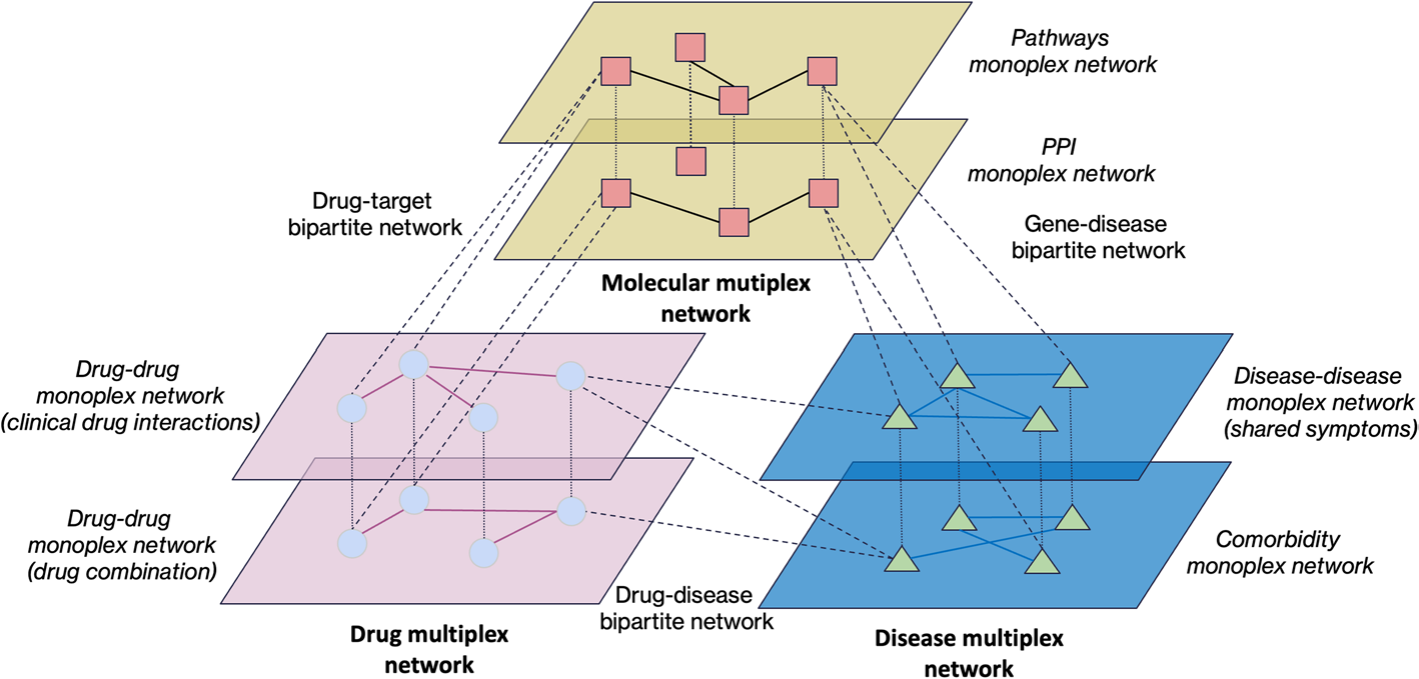
A universal multilayer network consisting of 3 distinct multiplex networks (gene, drug and disease), each represented by different colors (yellow, purple, and blue). Each of these multiplex networks consists of various types of nodes: squares are genes, circles are drugs and triangles are diseases. They are interconnected through three bipartite networks (gene-disease, drug-target and drug-disease), which are visualized here as bipartite interactions for clarity. The number of multiplex networks and layers inside the multiplex networks is arbitrary and could be more

In this work, we extended MultiVERSE [8], a multilayer network embedding algorithm tailored for the application of machine learning techniques to these multilayer networks but limited to a maximum of two multiplex networks. MultiVERSE is based on the VERSE framework [10], and coupled with Random Walks with Restart (RWR) on multilayer networks [9]. Recently, RWR has been extended to a universal random walk with restart using a method called MultiXrank [11] allowing the exploration of any kind of multilayer networks. We extended MultiVERSE with MultiXrank and it is now a universal multilayer network embedding method that we named MultiXVERSE. Our method can handle any multilayer network defined as a composition of various multiplex and monoplex networks interconnected through bipartite interaction networks (see Fig. 1 for an example). Within this multilayer structure, each network may also be weighted and/or directed. And we can add as many multiplex and bipartite networks as we want with this extension without limitations, except of course computational power.

Consequently, MultiXVERSE provides a means to network embedding on these multilayer networks, which are characterized by their rich and complex interactions. This approach is particularly effective in representing the multi-scale interactions typically observed in biological systems. For biology, this approach allows us to aggregate network data from drugs, diseases, genes, patients etc... in the same network representation and machine learning can be applied on the resulting embeddings for a wide variety of application including drug repositioning, new predicted gene-disease or drug-target links, the discovery of specific biological functional modules for diseases integrating genes and drugs etc... Several methods have been developed recently in AI and the mining of knowledge graphs [12–17]. To the best of our knowledge, it is the first time that network embedding can be applied to any kind of multilayer networks, including multiplex-heterogeneous networks, without any limitations on the number of multiplex networks or the type of neworks (weighted, directed, undirected).

In this article, we applied MultiXVERSE to a biological multilayer network containing data on gene, drug, and disease interactions and evaluated the quality of the embedding using link prediction (a standard approach in multilayer network embedding [8, 18, 19]). Second, we clustered the embeddings to find functional biological modules, which revealed new predictions of a link between GABA and cancer. Third, we applied link prediction to the embeddings of GABA agonists drugs and found new links between GABA receptors and cancer. Neurotransmitters are emerging targets in cancer [20, 21] and as such provide a case study particularly suited for evaluating our system. Finally, we experimentally tested the new prediction of GABA as a potential cause of cancer in a tadpole melanocyte model, and validated the prediction linking GABA-modulating drugs to a cancer-like phenotype in the absence of classic carcinogens, oncogenes, or DNA damage as the initiator of cellular conversion.

## Related prior work

We attempted to find similar methods in the literature, but unfortunately without success. Existing methods, such as PMNE [18], MVE [22], MNE [18], mvn2vec [23], DualHGNN [24], even though described sometimes under the category of multiplex heterogeneous networks, are only heterogeneous in the sense that they allow several types of edges linking the nodes; they can embed multiplex networks but can’t embed multiplex-heterogeneous networks such as as ours, e.g., composed of several types of nodes and edges linking same type of nodes and different types of nodes, and several bipartites. Also, most of the methods above rely on the manual construction of meta-paths, which limits their generality, which is not a limitation in the case of MultiXVERSE.

The closest methods we found are DualHGCN [25], FAME [26], GATNE [27], GTN [28], or MHGCN [29], which were developed for multiplex bipartite network embedding (we name it this way to distinguish it from our network), a network composed of several layers of bipartite networks. They can embed multiplex bipartite networks with several kind of edges between different types of nodes but can’t embed multiplex heterogeneous networks like ours that have in addition different types of edges between same types of nodes like (see our network representation in Fig. 1). The only similar method is MultiVERSE which is our previous implementation, having been quantitatively compared against state-of-the-art multiplex network embedding methods and a less general version of MultiXVERSE [8].

In our case, the term ‘multiplex heterogeneous network embedding’ includes significantly more type of networks than in our prior work. Other methods embed networks composed of several bipartites and having different types of nodes and different types of edges linking two different types of nodes. Our method embeds networks composed of different types of nodes and different types of edges linking different types of nodes too, but in addition we have different type of edges linking same type of nodes in the multiplex networks of our multiplex-heterogeneous network. Therefore, our new method can leverage more information.

We focused on the second meaning of multiplex-heterogeneous networks here, networks that are not simple to embed without compressing them into a single layer network or without important adaptations. If we do merge our network into a single layer for comparisons with heterogeneous network embedding methods, we lose a lot of information because the unique contribution of each layer to node relationships is lost. Some layers may be denser or more influential, leading to biases in the embedding where frequent connections overpower more subtle but meaningful ones. Also, in a multiplex-heterogeneous setting, some node types may have significantly more edges than others. If merged into a single layer, nodes with more connections will dominate the structure and distort the similarity metrics used for embedding.

## Materials and methods

### MultiXVERSE: a universal multilayer network embedding

We extended MultiVERSE [8] to MultiXVERSE for universal multilayer network embedding . This method computes the similarities between nodes using random walks with restart on any kind of multilayer network [11] and optimizes the embeddings using Kullback–Leibler minimization. We present the general method of MultiXVERSE in this section.

Within the MultiXVERSE framework, it is necessary to formulate a similarity metric for the multiplex-heterogeneous network, designated as $G_{MH}$. This metric, denoted $sim_{G} : V_{MH} \times V_{MH} \rightarrow {\mathbb {R}}$, maps pairs of nodes within $V_{MH}$ to a real number, reflecting their level of similarity. It is defined as follows:

1 $$\begin{aligned} \forall v \in V_{MH}, \ \sum _{u\in V} sim_{G} (v, u) = 1. \end{aligned}$$

Hence, $sim_G(v,.)$, which signifies the similarity metric for any given node $v$ within the multiplex-heterogeneous network $G_{MH}$, is conceptualized as a probability distribution. Given that, one can obtain the normalized similarity distribution within the embedding space by applying the softmax function. Formally, let $w_i$ represent the embedding of node $i$ within this space. Consequently, the similarity between the embeddings of two nodes $w_u$ and $w_v$ is characterized by the dot product $w_u \cdot w_v^T$, yielding the following expression:

2 $$\begin{aligned} sim_{Emb}(v,.) = \frac{exp(w_v \cdot w^T)}{\sum _{i=1}^n exp (w_v \cdot w_i)}. \end{aligned}$$

The purpose of MultiXVERSE is to closely estimate the similarity distribution within the embedding space, represented as $sim_{Emb}: V_{MH} \times V_{MH} \rightarrow {\mathbb {R}}$, such that for all $v$ in $V_{MH}$, the relationship $sim_G(v,.)$ is approximated by $sim_{Emb}(v,.)$. The optimization during the learning phase is executed through the minimization of the Kullback-Leibler divergence between the two similarity measures:

3 $$\begin{aligned} \sum _{v \in V_M} KL(sim_G(v,.)~\Vert ~ sim_{Emb}(v,.)) \end{aligned}$$

By keeping only the terms related to $sim_{Emb}$ as $sim_G$ is constant, we derive the objective function as follows:

4 $$\begin{aligned} {\mathcal {L}} = - \sum _{v \in V_M} sim_G(v,.)~log(sim_{Emb}(v,.)) \end{aligned}$$

At each iteration, since $sim_{Emb}$ is constructed as a softmax function, it necessitates normalization across the entire network’s nodes, a process that is computationally intensive. Analogous to the methodologies employed in the original MultiVERSE and VERSE algorithms, Noise Contrastive Estimation (NCE) is utilized to approximate these computations [30].

NCE trains a binary classifier to differentiate between node pairs sampled from the graph similarity distribution $sim_G$ and those obtained from a noise distribution *Q*.

We define *D* as a random variable denoting class labels, where $D=0$ if a node is sampled from the noise distribution *Q*, and $D=1$ if it is drawn from the empirical distribution. The expected value operator is denoted by ${\mathbb {E}}$. Given a node *u* sampled from ${\mathcal {P}}$ and another node *v* drawn from $sim_G (u, .)$, NCE generates $s < n$ negative samples $v_{neg}$ from the noise distribution *Q*(*u*).

Under this formulation, the objective function is expressed as the negative log-likelihood, which is minimized using logistic regression:

5 $$\begin{aligned} \begin{aligned} {\mathcal {L}}_{NCE}&= \sum _{\begin{array}{c} u \sim {\mathcal {P}} \\ v \sim sim_G(u,.) \end{array}} \Big [ \log P_W (D=1 ~|~ sim_{Emb}(u,v)) \\&+ s.{\mathbb {E}}_{v_{neg} \sim Q(u)} \log P_W(D=0 ~|~ sim_{Emb}(u, {\widetilde{v}})) \Big ] \end{aligned} \end{aligned}$$

where $P_W$ is computed using the sigmoid function $\sigma (x) = (1 + e^{-x})^{-1}$ applied to the dot product between embeddings $w_u$ and $w_v$. The similarity function $sim_{Emb}(u,.)$ is computed without normalization. It has been demonstrated that as *s* increases, the derivative of NCE approaches the gradient of cross-entropy, though in practice, small values are often sufficient [31]. Consequently, this approach effectively minimizes the KL-divergence between $sim_G$ and its learned representation.

In summary, VERSE offers a general framework for network embedding, with the primary requirement that $sim_G$ be a probability distribution. In this framework, the similarity $sim_G$ in the multiplex-heterogenous network is computed using MultiXrank [11]. And MultiXVERSE applies Kullback-Leibler minimization to optimize the embeddings. The parameters for random walks with restart on the multilayer networks are the following: $r = 0.7$, $\eta = \left[ \frac{1}{3}, \frac{1}{3}, \frac{1}{3}\right]$, $$\lambda = \begin{bmatrix} \frac{1}{3} & \frac{1}{3} & \frac{1}{3} \\ \frac{1}{3} & \frac{1}{3} & \frac{1}{3} \\ \frac{1}{3} & \frac{1}{3} & \frac{1}{3} \end{bmatrix}$$, $\delta _1 = 0.5$, $\delta _2 = 0.5$, $\delta _3 = 0.5$. and $$\tau = \left\{ \begin{bmatrix} \frac{1}{3}&\frac{1}{3}&\frac{1}{3} \end{bmatrix}, \begin{bmatrix} \frac{1}{2}&\frac{1}{2} \end{bmatrix}, \begin{bmatrix} \frac{1}{4}&\frac{1}{4}&\frac{1}{4}&\frac{1}{4} \end{bmatrix} \right\}$$.

The parameter r corresponds to the global restart probability. By default, we set this parameter to 0.7, which is a value often used in the literature [9]. The parameters $\eta$, $\lambda$, $\delta _1$, $\delta _2$, $\delta _3$, and $\tau$ in the MultiXrank framework define different probabilities governing transitions in the random walk with restart (RWR) on universal multilayer networks.

The parameter $\eta$ represents the probability of restarting the random walk within a given multiplex network, ensuring that the walker does not transition to another multiplex network at every step. The sum of all $\eta$ values across multiplex networks is constrained to equal 1. We assigned the same probability of restarting in each multiplex. The parameter $\lambda$ controls the probability of jumping between multiplex networks, allowing transitions across bipartite connections and ensuring that the walk spreads across the universal multilayer structure. We assigned the same probability of jumping in each multiplex.

The parameters $\delta _1$, $\delta _2$, and $\delta _3$ define the probability of moving between layers within the same multiplex network, thereby capturing layer-specific transition dynamics. In our case, the random walker explores several multiplex networks, the probability is defined as 0.5 for each multiplex; therefore, the random walker has the same probability to jump from one layer to another as to stay in the layer. The parameter $\tau$ governs the probability of restarting the random walk at a specific layer within a multiplex network, ensuring a balanced exploration of different layers. The sum of all $\tau$ values within a multiplex network must also sum to 1. Here, $\tau$ has been defined to assign the same probability of restarting in one layer of a specific multiplex network.

These parameters together regulate the random walk’s movement across layers, multiplex networks, and bipartite connections, ensuring a structured and controlled exploration of the multiplex-heterogeneous network.

RWR and NCE are known to be fast and efficient methods; the computational complexity is very suitable for nodes composed of millions of nodes as demonstrated here [8–11, 31]. The main computational limitation is likely to be the RWR part, as time complexity depends on the number of edges [11]. However, it has been demonstrated to be very practical for large networks with millions of nodes, something we don’t often have in biological datasets.

**Fig. 2:**
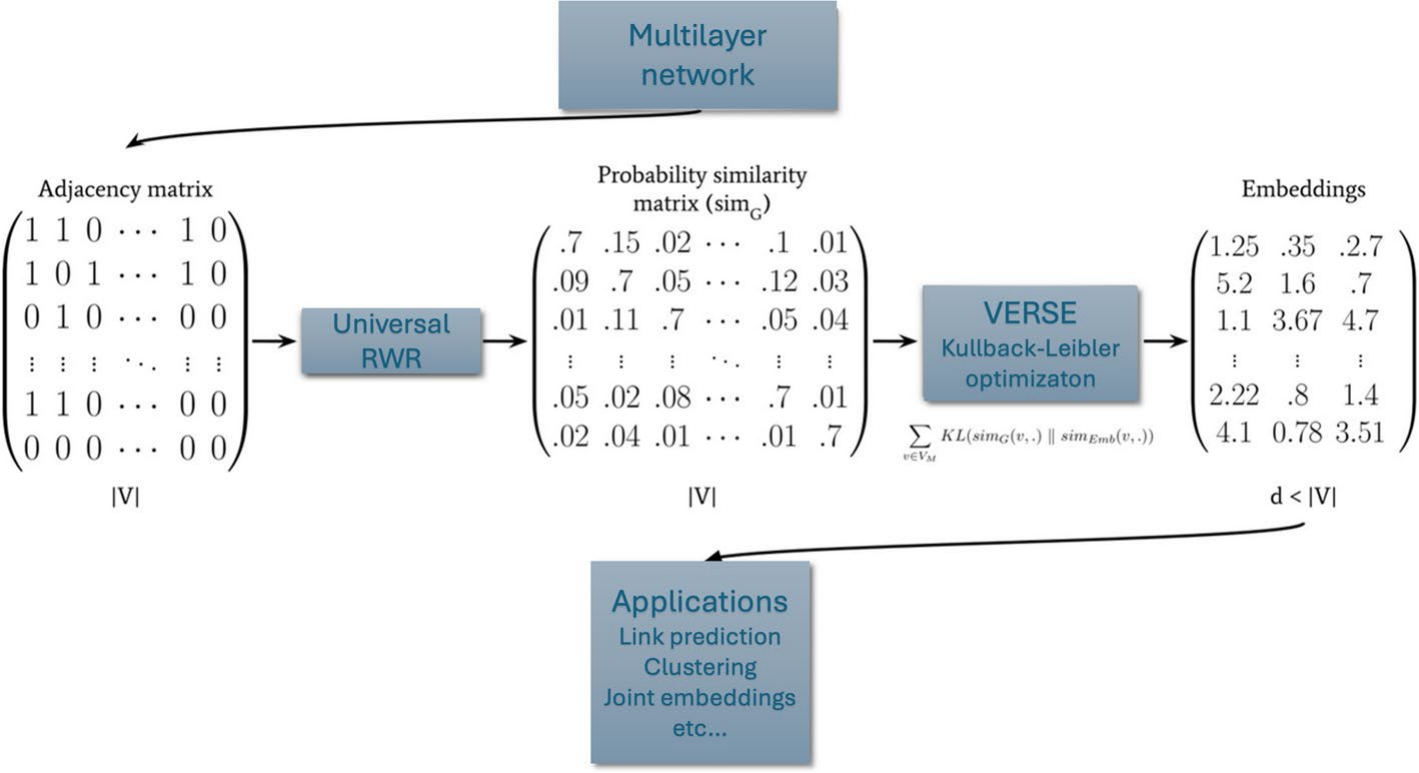
MultiXVERSE logical flow (figure adapted from [8]). We start with the adjacency matrix of the multilayer network; then, we apply universal random walk with restart to obtain the similarity matrix; and finally we apply the VERSE algorithm to compute the embeddings for diverse type of applications

The reader can refer to the original article for more details on the method and particular implementation of the algorithm [8] and [11]. The code for MultiXVERSE can be found at https://github.com/LPioL/MultiXVERSE. The general logical flow of the method can be found in Fig. 2.

#### Datasets for the gene-drug-disease multilayer network

We used several different datasets to construct the multiplex-heterogeneous network, which was composed of one human molecular multiplex network (3 layers), one drug multiplex network (4 layers) and one disease monoplex network. The multiplex networks are linked by 3 bipartite networks: drug-disease, gene-disease, and drug-target networks.

The multiplex networks are the following:

- Human molecular multiplex network: This network is a molecular network, extracted from [8], composed of 3 layers:
  1. The first layer is a protein-protein interaction (PPI) layer which integrates 4 datasets: Hi-Union, APID (apid.dep.usal.es) (Level 2, human only), Lit-BM (http://www.interactome-atlas.org/download).
  2. The second layer is a pathways layer constructed from the human Reactome data [32] extracted from NDEx [33].
  3. The third layer is a molecular complexes layer corresponding to the fusion of Hu.map [34] and Corum [35].
- Drug multiplex network: The multiplex drug network integrates several sources and interaction types and has been extracted from [11]. Data derived from Cheng et al. [36] and the pharmacological drugs interaction network available at snap.stanford.edu were utilized. In this network, drugs are named according to DrugBank conventions, encompassing both the multiplex network and its associated bipartite networks:
  1. The first layer corresponds to clinical drug interactions. It includes 14,822 clinically reported adverse drug-drug interactions among 667 drugs.
  2. The second is the experimental drug combinations layer. It contains 737 experimentally validated drug combinations involving 376 drugs.
  3. The third represents the predicted drug combinations and includes 2,080 network-predicted combinations for hypertensive drugs, covering 65 different drugs.
  4. The last layer of the drug multiplex network includes the pharmacologic drug-drug interactions and consists of 48,514 interactions determined by the pharmacological effects of one drug on another, involving 1,514 drugs.
- Disease network: The Disease multiplex network (DIS) has been structured into two layers, each representing different aspects of disease relationships:
  1. Disease-Disease Network Based on Shared Symptoms: Originating from a bipartite disease-symptoms network [37], this layer forms connections based on the cosine distance between diseases, retaining all interactions where this distance is above 0.5, indicating significant symptom overlap.
  2. Comorbidity Network: This layer integrates epidemiological data from [38] to illustrate the comorbidity relationships among diseases, highlighting epidemiological correlations.
  Each layer provides a unique perspective on disease interactions, encompassing treatment similarities, symptom relationships, and epidemiological data. .

The bipartite networks are:

- Gene-Disease Network: We extracted the curated gene-disease bipartite network from the DisGeNET database in order to connect the two molecular and disease multiplex networks.
- Drug-target Network: This network combines drug-target associations from multiple sources including DrugBank Release Version 5.1.8 (accessible at https://go.drugbank.com/releases/latest), DrugCentral release v10.12 (available at https://drugcentral.org/download), and associations described by Cheng et al [36].
- Disease-Drug Network: The associations between diseases and drugs are obtained from the Comparative Toxicogenomics Database.

#### Evaluation of the approach

**Link prediction**

Like previous studies [8, 18, 19, 39] and as we didn’t find in the literature any method capable of computing the embedding of such a network, we decided to evaluate the quality of the embeddings using link prediction and in addition to test the predictions of our model experimentally. We employed link prediction to assess the efficacy of our embeddings and validate our universal multilayer network embedding method for network biology and medicine. Our link prediction methodology entailed initially removing 30$\%$ of bipartite edges randomly in each bipartite network to form a training multilayer network. Subsequently, we employed a Random Forest classifier to this training network, as described in [8], and performed evaluations on a withheld subset consisting of 30$\%$ of the edges. We repeated this evaluation protocol 10 times. The binary classifier’s training method included the utilization of various operators on the node embeddings. These operators comprised Hadamard, Weighted-L1, Weighted-L2, Average, and cosine.

The objective of this validation method was to ascertain the quality of the embeddings in the discovery of novel drug-gene-disease associations. At present, conducting direct comparative analyses with alternative methodologies is not practicable due to the unique nature of the embedding process for multilayer networks with three distinct node types from 3 different multiplex networks, a feature not yet paralleled in the existing literature.

**Case study on cancer and neurotransmitters**

The second approach we used for validation was to test the method on a case study, here to assess the link between neurotransmitter and cancer [40–42]. Serotonin has already been linked to cancer [40–42] and we wanted to know if other neurotransmitters might be predicted by our model which could lead to the discovery of new targets and drug repositioning for cancer.

In order to test our system on these new results, we focused on biological modules. Once MultiXVERSE had been applied to the drug-disease-gene multilayer network, we used a clustering method on the embeddings and analyzed the clusters. The clustering method we applied was spherical k-means [43] with $k=500$ applied on the embedding.

We then applied link prediction to GABA agonist drugs using a Random Forest classifier with the operator ’Average’.

### Experimental testing:materials and method

#### Animal husbandry

Animal care was done in compliance with, and approval from, the Institutional Animal Care and Use Committee (IACUC) under protocol number M2023-18 of Tufts University. Xenopus embryos were collected according to standard protocols [44] in 0.1X MMR ((Marc’s Modified Ringers) pH 7.8 + 0.1$\%$ Gentamicin. Xenopus embryos were staged according to [45]. All experiments were approved by the University Institutional Animal Care and Use Committee (IACUC) under the protocol number M2023-18. We have complied with all relevant ethical regulations for animal use. Xenopus Laevis embryos were fertilized in vitro according to standard protocols, from eggs obtained from the adult frogs living in our Xenopus facility.

#### Drug exposure

Stocks of muscimol (Tocris 0289) were kept at 10 mM concentration in DMSO. Embryos were exposed in 0.1X MMR during stages 12-43 in muscimol at a final concentration of 50 $\mu$M.

#### Histology

Embryos at stage 43-45 were embedded in JB4 according to the manufacturer’s directions (Polysciences), and sectioned on a Leica microtome at 20$\mu$. They were then photographed on a Nikon SMZ-1500 microscope.

## Results

### Computational results

#### Evaluation results using link prediction

The ROC-AUC is superior to 0.9 with Average operators for all bipartite networks (see Table 1), meaning that the method can predict with high precision the removed 30% of gene-disease, drug-disease and drug-target links from the corresponding multiplex-heterogeneous networks.

**Table 1:** ROC-AUC scores for link prediction using MultiXVERSE. Link predictions are computed for the bipartite interactions of the multiplex-heterogeneous networks. We applied our evaluation protocol 10 times and found ROC-AUC superior to 0.9 with Average operators for all bipartite networks. The scores higher than 0.9 are highlighted in bold

	ROC-AUC			
**Operators**	Gene-disease	Drug-target	Drug-disease	Average
Hadamard	$0.88 \pm 0.002$	$\mathbf {0.92 \pm 0.001}$	$0.88 \pm 0.003$	$\mathbf {0.90 \pm 0.005}$
Weighted_L1	$0.88 \pm 0.002$	$0.62 \pm 0.01$	$0.77 \pm 0.002$	$0.76 \pm 0.005$
Weighted_L2	$0.88 \pm 0.003$	$0.63 \pm 0.01$	$0.76 \pm 0.001$	$0.76 \pm 0.004$
Average	$\mathbf {0.94 \pm 0.002}$	$\mathbf {0.93 \pm 0.003}$	$\mathbf {0.91 \pm 0.002}$	$\mathbf {0.93 \pm 0.002}$
Cosine	$0.55 \pm 0.005$	$0.83 \pm 0.004$	$0.70 \pm 0.002$	$0.70 \pm 0.004$

The variance across all operators is minimal, indicating that the network embedding method demonstrates high robustness and consistency in each iteration of the link prediction evaluation test.

#### Consistency of MultiXVERSE with MultiVERSE results on the progeria cluster

**Fig. 3:**
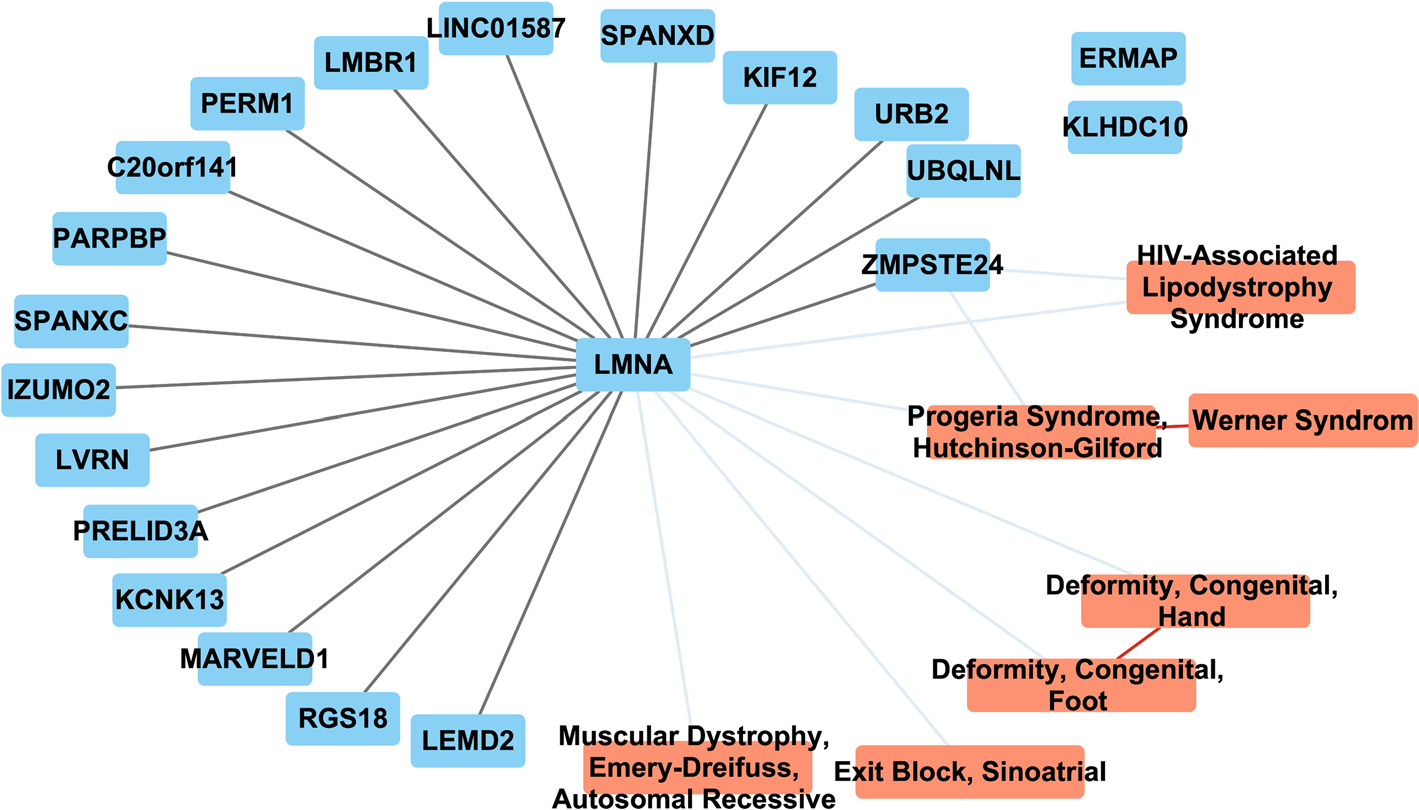
Network representation of the progeria cluster. Blue, orange are respectively genes, diseases. Black, light blue, and red links are respectively molecular multiplex, gene-disease, and disease multiplex links

To assess the quality of the clustering of our embeddings, we analyzed the progeria cluster similarly to [8] (see Fig. 3). Hutchinson-Gilford Progeria Syndrome (HGPS) is a rare genetic disorder that causes premature aging. It is characterized by symptoms such as slowed postnatal growth, facial structural abnormalities, premature cardiovascular diseases, lipodystrophy, hair loss, and widespread osteodysplasia. HGPS arises from mutations in the LMNA genes, leading to the production of a deleterious version of the Lamin A protein, known as Progerin.

The results are similar to those obtained with MultiVERSE: LMNA and HGPS were both found to be associated. We found several genes in both clusters including ZMPSTE24 in the cluster that is associated to accelerated aging in the literature and LMNA [46, 47], but also LEMD2, RGS18, MARVELD1, KCNK13, IZUMO2, PERM1, LINC01857, and KIF12n. The clusters share diseases including muscular dystrophy, the Werner syndrome (the adult premature aging syndrome), deformities of the hand and foot, and cardiac disease associated with progeria [48].

KCNK13 is an especially interesting gene and encodes a $\hbox {K}^+$ potassium ion channel (Potassium Two Pore Domain Channel Subfamily K Member 13). It is related to the Birk-Barel syndrome (BIBARS) - a rare genetic disorder characterized by motor and speech delay, impaired intellectual development, early feeding difficulties, muscular hypotonia, hyperactivity, aggression, and facial dysmorphism. This syndrome shares part of its phenotype with HGPS. HGPS has also been related to bioelectricity [49] which can fall under the context of aging (or premature aging) as a channelopathy [50].

Therefore, we conclude that we have similar results to the previous version of MultiVERSE [8], even if we have more multiplex and bipartite networks (MultiVERSE has been applied on a gene-disease mutilayer network) and a different set of networks, showing a good robustness to the integration of new data.

#### The clustering of the embeddings shows serotonin and GABA pathways linked to cancer and developmental disorder

**Fig. 4:**
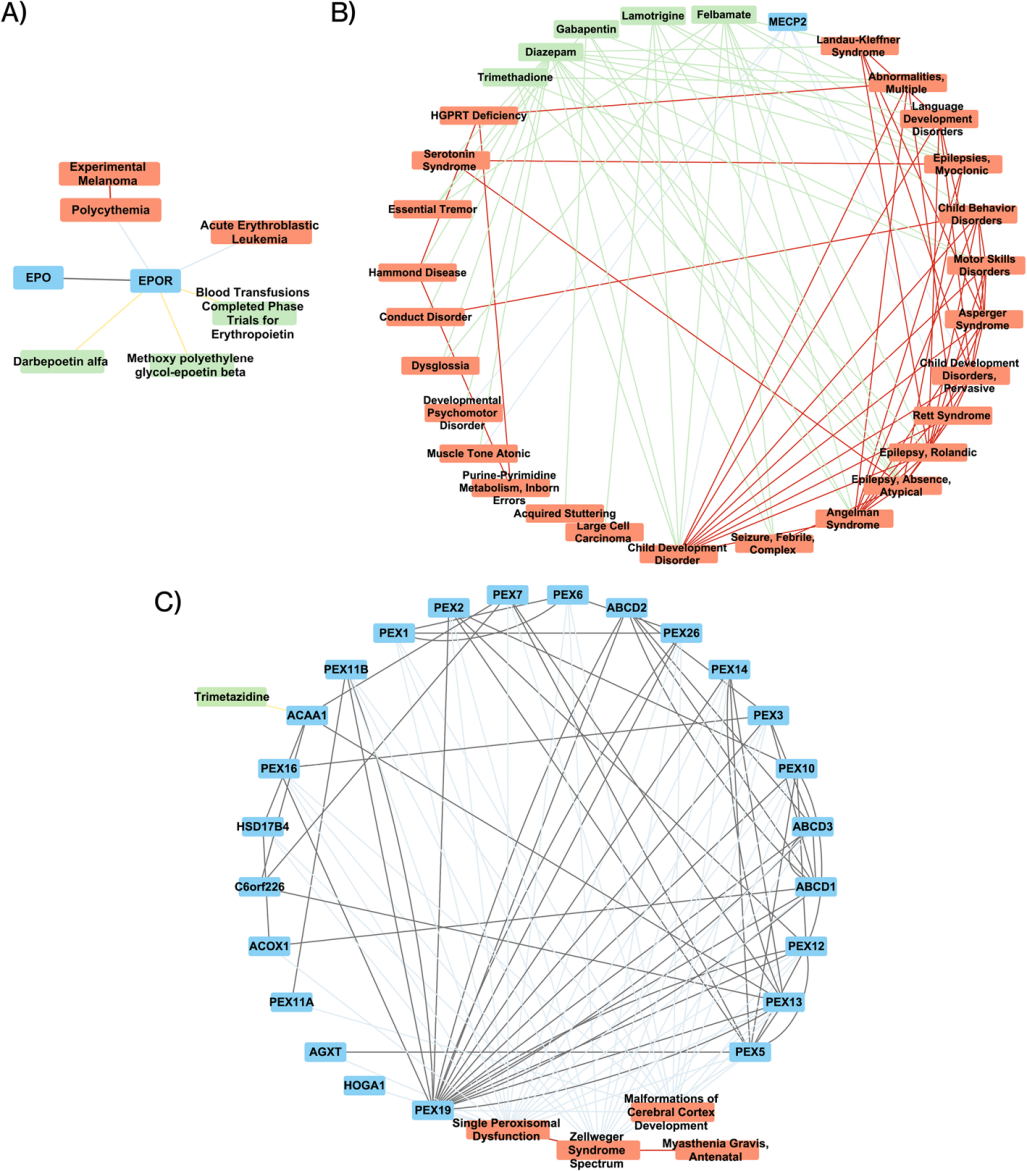
Network representations of the clusters integrating cancer or developmental disorders and neurotransmitters. A) Network cluster linking drugs regulating GABA and melanoma. B) Network cluster linking GABA drugs like gabapentin and large cell carcinoma. C) Network cluster linking trimetazidine and malformations of cortex development. Blue, orange and green boxes are respectively genes, diseases and drugs. Black, light blue, yellow, light green and red links are respectively molecular multiplex, gene-disease, drug-target, drug-disease, and disease multiplex links

In order to learn more about the link between cancer, developmental disorders and neurotransmitters [40–42], we analyzed the different clusters integrating those three components. We found that several clusters show a link between neurotransmitters including GABA and serotonin with cancer of malformations. One cluster (see Fig. 4A) includes EPO and Darbepoetin alpha. Recombinant human erythropoietin is commonly used in clinical settings to treat anemia associated with cancer and chemotherapy. However, recent clinical trials indicate that rhEPO might also negatively affect disease progression and patient survival [51]. Interestingly, EPO is known to increase GABA currents [52] suggesting an implication of GABA neurotransmitter in the adverse effect of EPO in cancer development.

A second cluster is linking developmental disorders, neurotransmitter drugs (gabapentin with lamotrigine) with serotonin syndrome and large cell carcinomas (see Fig. 4B). Gabapentin is a structural analogue of the inhibitory neurotransmitter gamma-aminobutyric acid (GABA) [53]. Lamotrigine is an anti-glutamate agent and may enhance GABAergic transmission [54]. Lamotrigine can also augment serotonin re-uptake inhibitors [55]. This cluster suggests a link between GABA, serotonin and cancer, and that has been recently studied [40–42].

Lastly, we found a cluster (see Fig. 4C) including developmental disorders and Trimetazidine, which is an anti-ischemic drug that can inhibit platelet aggregation and regulate the expression of serotonin in a rodent model [56].

These results indicate GABA pathways as potentially implicated in cancer development in addition to serotonin pathways [40–42].

Our model predicted links between cancer and serotonin and GABA via clustering, and the serotonin link has been validated by published data [40–42]. We decided to test the link between GABA and cancer using link prediction. We found in the literature [57, 58] that there was a recent known link between GABA and cancer, drawn from analysis of clinical samples and functional data in vitro [59, 60].

#### GABA drugs show different types of cancer in the first 10 predictions

The link between GABA and cancer has been studied before and it has been found that GABA has a driver role in controlling stem and proliferative cell state through GHB production in glioma [61]. Membrane potential and GABA(A) receptor expression differences have been found between hepatic tumor versus non-tumor stem cells [62]. However, contradictory evidence has been reported showing that GABA could have an inhibitory effect on tumor progression or cell proliferation [63, 64]. Given this contradictory evidence, we decided to validate further this link in our system.

We applied link prediction using the embeddings on Baclofen, Zaleplon, Clobazam, Progabide, Zolpidem and Gabapentin. These drugs are GABA agonists [65–69].

All of the tested drugs showed a link with cancer in the 10 first predictions, with the exception of Zolpidem which showed a link with rectum neoplasm at the 18th prediction. Baclofen had a prediction for adrenal cortical carcinoma, Zaplelon for cancer of the esophagus and adrenal cortical carcinoma, Clobazam for soft tissue sarcoma and experimented neoplasm, Progabide for soft tissue neoplasm, and Gabapentin for soft tissue neoplasm.

The new prediction of GABA agonist as a potential trigger of cancer had not yet been validated; thus, we sought to test it experimentally.

### Experimental results

**Fig. 5:**
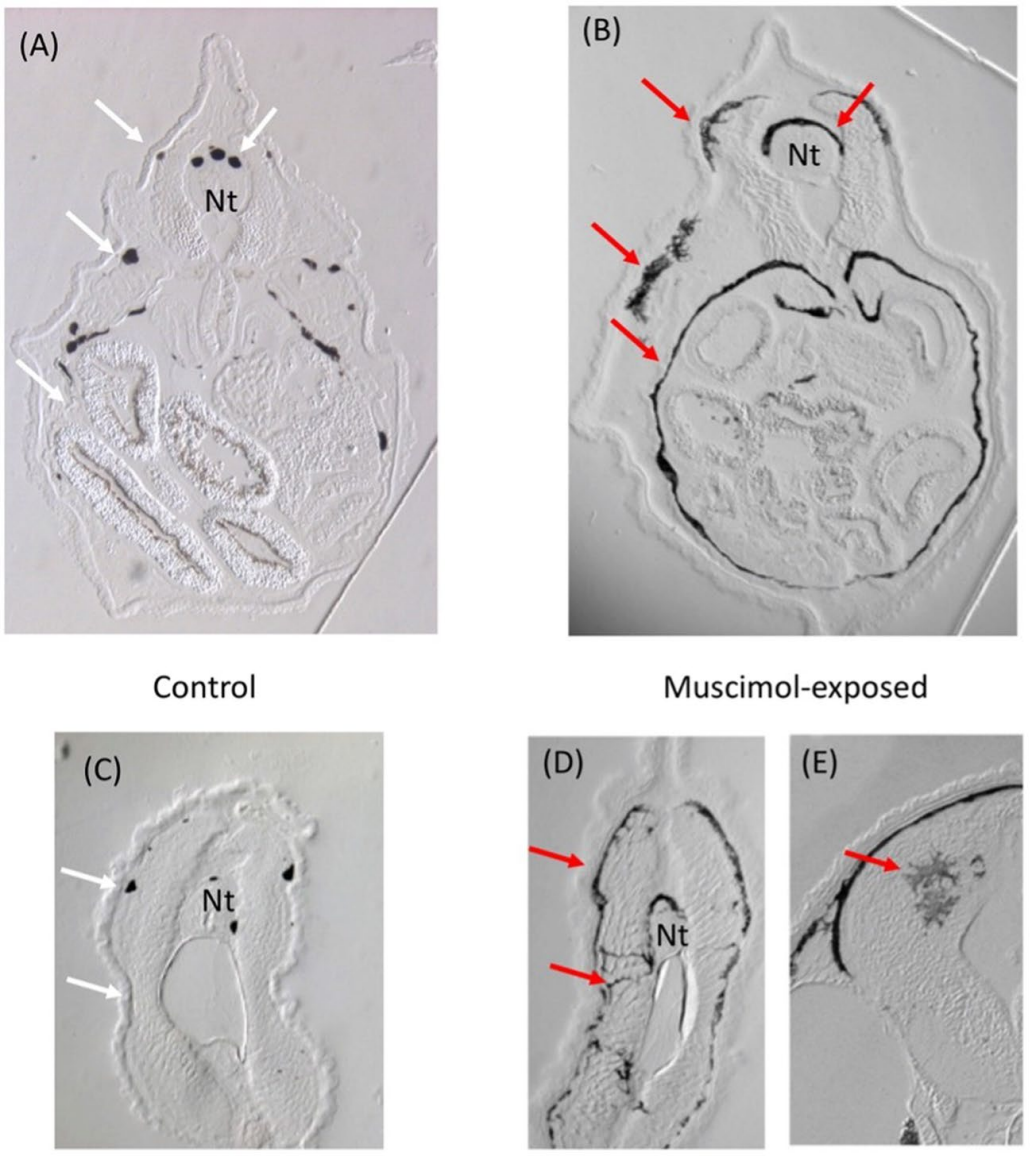
Melanocyte conversion phenotype induced by muscimol exposure. All panels are transverse sections; A,B are taken through the gut, while C,D are taken through the mid-tail. (A) Control embryos show small numbers of discrete, round melanocytes (white arrows). (B) In contrast, muscimol exposure induces melanocytes to over-proliferate and form long, stretched out projections that cover the gut cavity and other locations (red arrows). Compared to round melanocytes in the trunk and tail of controls (C), melanocytes in muscimol-treated animals (D) can be clearly seen to have an abnormal invasive shape and distribution (D). (E) Closeup of melanocytes invading the neural tube after muscimol exposure. Nt = neural tube

In order to test the prediction that GABA pathway modulation should functionally induce a cancer-like phenotype in vivo, we used larval Xenopus laevis — a powerful model system commonly used to understand cancer-like dysregulation of cell function [70–72]. One type of cancer-related phenotype that is especially readily investigated in frog embryos is the conversion of melanocytes, from normal pigment cells to hyper-proliferative, invasive melanoma-like behavior and induction of cancer markers [40, 42, 72, 73]. While normal melanocytes are also migratory during development, activating them via bioelectric dysregulation of chloride channel function induces a completely different phenotype [40, 73].

In order to perturb GABA signaling, we used muscimol — a well-known GABA agonist [74–78]. Muscimol itself is not in the original data we used in our model but it is a GABA(A) agonist like Progabide [79, 80] that was found in the link prediction (see above), enabling us to test the utility of the model’s categorical predictions for novel drugs that it did not have direct experience with. Fifty Xenopus embryos, in triplicate, were exposed to 50 $\mu$M muscimol between stage 12 to stage 45 (after completion of gastrulation through swimming tadpole stages), and then sectioned. The results are shown in Fig. 5; this was an extremely consistent and highly penetrant phenotype observed in 100% of the treated animals macroscopically, and in 10 sectioned (randomly-selected) animals. In contrast to controls, all of the exposed animals exhibited a drastic hyperpigmentation due to the melanocytes’ changing shape (from their normal round form to a much more elongated morphology), and migrating into inappropriate regions that are normally clear. This phenotype has previously been characterized quantitatively with respect to melanocyte shape and number, as well as the expression of cancer-related markers and melanoma-like migration into gut, brain, and vascular tissues [40–42, 72, 73]. These results confirm the prediction of the model and link GABA-modulating drugs to a cancer-like phenotype in the absence of classic carcinogens, oncogenes, or DNA damage as the initiator of cellular conversion.

## Discussion

In this work, we presented what is, to the best of our knowledge, the first universal multiplayer network embedding method with no limitations on number of multiplex and bipartite networks, thanks to recent developments in RWR [11]. We applied MultiXVERSE to a multilayer network containing gene, drug, and diseases interactions and evaluated the quality of the embeddings using both clustering and link prediction. Our model predicted links between cancer, serotonin, and GABA via clustering. The serotonin link has been validated by published data [40–42]; likewise, the GABA link has been studied before, suggesting that GABA has a driver role in controlling stem and proliferative cell state through GHB production in glioma [61]. Also reported have been membrane potential differences and GABA(A) receptor expression in hepatic tumor versus non-tumor stem cells [62]. However, contradictory evidence has been reported showing that GABA could have an inhibitory effect on tumor progression or cell proliferation [63, 64]. We tested the prediction from clustering between GABA and cancer and confirmed in vivo a causal link between GABA and a cancer-like phenotype in a vertebrate model system in vivo, in the absence of classic carcinogens, oncogenes, or DNA damage as the initiator of cellular conversion. These kinds of data have important implications for understanding of cancer etiology and possible normalization [81–83] efforts using neurotransmitter modulators, and need to be tested in preclinical mammalian models next. More research is necessary to understand the impact of GABA on cancer.

Our main domain of application here was drug discovery for cancer, but the embeddings of gene-drug-disease multilayer networks may be used for many other applications. Different questions in network biology could be addressed by finding new genes related to specific diseases, such as the interesting target - the ion channel KCNK13 - revealed by our case study on the progeria cluster. This may be especially relevant due to the recent hypotheses about the role of bioelectric signaling in aging [50, 84, 85]. We also applied link prediction for different drug-disease associations. Our method for drug repurposing has currently one significant limitation: in the case of new drug-target predictions, we don’t know if the drug will activate or inactivate the target. This may be resolved by using directed networks (unfortunately still rare) in the multiplex networks and training link prediction models on the embedding integrating the directional information of the links. It could be interesting too to add neighborhood-level structural representation for predicting new links in the networks, similarly to [86], or add attention mechanisms for drug repositioning [87, 88].

The model’s interpretability could be enhanced to better elucidate the decision-making process and underlying biological mechanisms. This is a theoretical problem that includes a large part of the field of machine learning - (neural) network interpretability [89–92]. We focus here more on the biological usability of the method and empirical results, which will be a good addition to the field of network interpretability in biology and AI as a whole. However, we have to emphasize that the interpretability of our method probably does not have the same degree of complexity of deep learning, which gives cause for optimism for interpretability in the future.

To improve the capabilities of Large Language Models (LLMs) in processing text-enriched images, researchers have developed embeddings specifically designed to capture image contexts. These embeddings are integrated as soft prompt inputs in LLMs, enhancing the models’ ability to effectively handle visual information [93]. Similarly, we could use the node embeddings as input to use the power of LLMs for generative drug discovery including the richness of multiplex-heterogeneous network biological data. One relevant effort that can be integrated into this framework in the future is the bioinformatics of shape, which seeks to formalize and make amenable to machine learning data on large-scale anatomical outcomes in embryogenesis, regeneration, cancer, and bioengineering [94, 95]. We expect that future systems that combine pattern inference with diverse multi-modal datasets, comprising physiological, anatomical, and molecular-biological data will be a critical aid to human scientists and clinicians seeking to develop interventions for a wide range of biomedical applications.

## Conclusion

The development of MultiXVERSE represents a significant advancement in the integration and analysis of multilayer networks for biological research. By providing a universal, scalable framework for multilayer network embedding, MultiXVERSE enables the systematic exploration of molecular and phenotypic interactions across diverse biological contexts. Our experimental validation of the predicted link between GABA and cancer using Xenopus laevis underscores its capability to generate biologically meaningful hypotheses and accelerate breakthroughs in multi-omics research.

Future directions include applying MultiXVERSE to additional multi-omics datasets and integrating it with high-throughput experimental pipelines for systematic hypothesis generation and validation, particularly in drug discovery. Beyond its biological applications, MultiXVERSE is a versatile tool that can be utilized for analyzing multilayer networks in a wide range of fields, including social sciences and other complex systems. By offering a universal framework, MultiXVERSE paves the way for novel insights and interdisciplinary collaborations in multilayer network research.

## Data Availability

All relevant data are in the manuscript. No new materials were generated in this study. We used several different datasets to construct the multiplex-heterogeneous network, which was composed of one human molecular multiplex network (3 layers), one drug multiplex network (4 layers) and one disease monoplex network. The multiplex networks are linked by 3 bipartite networks: drug-disease, gene-disease, and drug-target networks. The multiplex networks are the following: $\bullet$ Human molecular multiplex network: This network is a molecular network, extracted from [8], composed of 3 layers: [1.] The first layer is a protein-protein interaction (PPI) layer which integrates 4 datasets: Hi-Union, APID (apid.dep.usal.es) (Level 2, human only), Lit-BM (http://www.interactome-atlas.org/download). [2.] The second layer is a pathways layer constructed from the human Reactome data [32] extracted from NDEx [33]. [3.] The third layer is a molecular complexes layer corresponding to the fusion of Hu.map [34] and Corum [35]. $\bullet$ Drug multiplex network: The multiplex drug network integrates several sources and interaction types and has been extracted from [11]. Data derived from Cheng et al. [36] and the pharmacological drugs interaction network available at snap.stanford.edu were utilized. In this network, drugs are named according to DrugBank conventions, encompassing both the multiplex network and its associated bipartite networks: $\bullet$ Disease network: The Disease multiplex network (DIS) has been structured into two layers, each representing different aspects of disease relationships: [1.] Disease-Disease Network Based on Shared Symptoms: Originating from a bipartite disease-symptoms network [37], this layer forms connections based on the cosine distance between diseases, retaining all interactions where this distance is above 0.5, indicating significant symptom overlap. [2.] Comorbidity Network: This layer integrates epidemiological data from [38] to illustrate the comorbidity relationships among diseases, highlighting epidemiological correlations.

## References

[1] Kivelä M, Arenas A, Barthelemy M, Gleeson JP, Moreno Y, Porter MA. Multilayer networks. J Complex Netw. 2014;2(3):203–71.

[2] Zitnik M, Li MM, Wells A, Glass K, Gysi DM, Krishnan A, Murali T, Radivojac P, Roy S, Baudot A, et al. Current and future directions in network biology, arXiv preprint arXiv:2309.08478, 2023. 10.1093/bioadv/vbae099 PMC11321866 39143982

[3] Liao L, He X, Zhang H, Chua T-S. Attributed social network embedding. IEEE Trans Knowl Data Eng. 2018;30(12):2257–70.

[4] Ma G, Lu C-T, He L, Philip SY, Ragin AB. Multi-view graph embedding with hub detection for brain network analysis. In 2017 IEEE International Conference on Data Mining (ICDM), pp. 967–972, IEEE, 2017.

[5] Csermely P, Kunsic N, Mendik P, Kerestély M, Faragó T, Veres DV, Tompa P. Learning of signaling networks: molecular mechanisms. Trends Biochem Sci. 2020;45(4):284–94. 32008897 10.1016/j.tibs.2019.12.005

[6] Kovács IA, Mizsei R, Csermely P. A unified data representation theory for network visualization, ordering and coarse-graining. Sci Rep. 2015;5(1):13786. 26348923 10.1038/srep13786 PMC4642569

[7] Nelson W, Zitnik M, Wang B, Leskovec J, Goldenberg A, Sharan R. To embed or not: network embedding as a paradigm in computational biology, *Frontiers in genetics*, **10**, 2019. 10.3389/fgene.2019.00381 PMC6504708 31118945

[8] Pio-Lopez L, Valdeolivas A, Tichit L, Remy É, Baudot A. Multiverse: a multiplex and multiplex-heterogeneous network embedding approach. Sci Rep. 2021;11(1):1–20. 33888761 10.1038/s41598-021-87987-1 PMC8062697

[9] Valdeolivas A, Tichit L, Navarro C, Perrin S, Odelin G, Levy N, Cau P, Remy E, Baudot A. Random walk with restart on multiplex and heterogeneous biological networks. Bioinformatics. 2018;35(3):497–505. 10.1093/bioinformatics/bty637 30020411

[10] Tsitsulin A, Mottin D, Karras P, Müller E. Verse: Versatile graph embeddings from similarity measures. In Proceedings of the 2018 World Wide Web Conference, pp. 539–548, International World Wide Web Conferences Steering Committee, 2018.

[11] Baptista A, Gonzalez A, Baudot A. Universal multilayer network exploration by random walk with restart. Commun Phys. 2022;5(1):170.

[12] Bang D, Lim S, Lee S, Kim S. Biomedical knowledge graph learning for drug repurposing by extending guilt-by-association to multiple layers. Nat Commun. 2023;14(1):3570. 37322032 10.1038/s41467-023-39301-y PMC10272215

[13] Middleton L, Melas I, Vasavda C, Raies A, Rozemberczki B, Dhindsa RS, Dhindsa JS, Weido B, Wang Q, Harper AR, et al. Phenome-wide identification of therapeutic genetic targets, leveraging knowledge graphs, graph neural networks, and uk biobank data, Science Advances, **10**(19), p. eadj1424, 2024. 10.1126/sciadv.adj1424 PMC11078195 38718126

[14] Huang K, Chandak P, Wang Q, Havaldar S, Vaid A, Leskovec J, Nadkarni GN, Glicksberg B. S, Gehlenborg N, Zitnik M. A foundation model for clinician-centered drug repurposing, Nature Medicine, pp. 1–13, 2024. 10.1038/s41591-024-03233-x PMC11645266 39322717

[15] Li MM, Huang Y, Sumathipala M, Liang MQ, Valdeolivas A, Ananthakrishnan AN, Liao K, Marbach D, Zitnik M. Contextual ai models for single-cell protein biology. Nat Methods. 2024;21(8):1546–57. 39039335 10.1038/s41592-024-02341-3 PMC11310085

[16] Hu Y, Oleshko S, Firmani S, Zhu Z, Cheng H, Ulmer M, Arnold M, Colomé-Tatché M, Tang J, Xhonneux S, et al. Path-based reasoning for biomedical knowledge graphs with biopathnet, bioRxiv, 2024. 10.1038/s41551-025-01598-z PMC13472943 41559192

[17] Jiménez A, Merino MJ, Parras J, Zazo S. Explainable drug repurposing via path based knowledge graph completion. Sci Rep. 2024;14(1):16587. 39025897 10.1038/s41598-024-67163-x PMC11258358

[18] Zhang H, Qiu L, Yi L, Song Y. Scalable multiplex network embedding. IJCAI. 2018;18:3082–8.

[19] Bagavathi A, Krishnan S, Multi-net: A scalable multiplex network embedding framework. In International Conference on Complex Networks and their Applications, pp. 119–131, Springer, 2018.

[20] Jiang S-H, Hu L-P, Wang X, Li J, Zhang Z-G. Neurotransmitters: emerging targets in cancer. Oncogene. 2020;39(3):503–15. 31527667 10.1038/s41388-019-1006-0

[21] Mancusi R, Monje M. The neuroscience of cancer. Nature. 2023;618(7965):467–79. 37316719 10.1038/s41586-023-05968-y PMC11146751

[22] Qu M, Tang J, Shang J, Ren X, Zhang M, Han J. An attention-based collaboration framework for multi-view network representation learning. In Proceedings of the 2017 ACM on Conference on Information and Knowledge Management, pp. 1767–1776, 2017.

[23] Shi Y, Han F, He X, He X, Yang C, Luo J, Han J. mvn2vec: preservation and collaboration in multi-view network embedding,” arXiv preprint arXiv:1801.06597, 2018.

[24] Liao J, Yan J, Tao Q. Dualhgnn: A dual hypergraph neural network for semi-supervised node classification based on multi-view learning and density awareness. In 2023 International Joint Conference on Neural Networks (IJCNN), pp. 1–10, IEEE, 2023.

[25] Xue H, Yang L, Rajan V, Jiang W, Wei Y, Lin Y. Multiplex bipartite network embedding using dual hypergraph convolutional networks. Proc Web Conf. 2021;2021:1649–60.

[26] Liu Z, Huang C, Yu Y, Fan B, Dong J. Fast attributed multiplex heterogeneous network embedding. In Proceedings of the 29th ACM International Conference on Information & Knowledge Management, pp. 995–1004, 2020.

[27] Cen Y, Zou X, Zhang J, Yang H, Zhou J, Tang J. Representation learning for attributed multiplex heterogeneous network. In Proceedings of the 25th ACM SIGKDD international conference on knowledge discovery & data mining, pp. 1358–1368, 2019.

[28] Yun S, Jeong M, Kim R, Kang J, Kim HJ. Graph transformer networks. Adv Neural Inf Proc Syst, **32**, 2019.

[29] Fu C, Yu P, Yu Y, Huang C, Zhao Z, Dong J. Mhgcn+: multiplex heterogeneous graph convolutional network. ACM Trans Intell Syst Technol. 2024;15(3):1–25.

[30] Gutmann M, Hyvärinen A. Noise-contrastive estimation: A new estimation principle for unnormalized statistical models. In Proceedings of the Thirteenth International Conference on Artificial Intelligence and Statistics, pp. 297–304, 2010.

[31] Mnih A, Kavukcuoglu K. Learning word embeddings efficiently with noise-contrastive estimation. Advances in neural information processing systems, vol. 26, 2013.

[32] Croft D, Mundo AF, Haw R, Milacic M, Weiser J, Wu G, Caudy M, Garapati P, Gillespie M, Kamdar MR, et al. The reactome pathway knowledgebase. Nucleic Acids Res. 2014;42(D1):D472–7. 24243840 10.1093/nar/gkt1102 PMC3965010

[33] Pratt D, Chen J, Welker D, Rivas R, Pillich R, Rynkov V, Ono K, Miello C, Hicks L, Szalma S, et al. Ndex, the network data exchange. Cell Syst. 2015;1(4):302–5. 26594663 10.1016/j.cels.2015.10.001 PMC4649937

[34] Drew K, Lee C, Huizar RL, Tu F, Borgeson B, McWhite CD, Ma Y, Wallingford JB, Marcotte E.M. Integration of over 9,000 mass spectrometry experiments builds a global map of human protein complexes. Molecular Syst Biol, **13**(6), 2017. 10.15252/msb.20167490 PMC5488662 28596423

[35] Giurgiu M, Reinhard J, Brauner B, Dunger-Kaltenbach I, Fobo G, Frishman G, Montrone C, Ruepp A. Corum: the comprehensive resource of mammalian protein complexes-2019. Nucleic Acids Res. 2019;47(D1):D559–63. 30357367 10.1093/nar/gky973 PMC6323970

[36] Cheng F, Kovács IA, Barabási A-L. Network-based prediction of drug combinations. Nat Commun. 2019;10(1):1–11. 30867426 10.1038/s41467-019-09186-x PMC6416394

[37] Zhou X, Menche J, Barabási A-L, Sharma A. Human symptoms-disease network. Nat Commun. 2014;5(1):1–10. 10.1038/ncomms5212 24967666

[38] Jensen AB, Moseley PL, Oprea TI, Ellesøe SG, Eriksson R, Schmock H, Jensen PB, Jensen LJ, Brunak S. Temporal disease trajectories condensed from population-wide registry data covering 6.2 million patients. Nat Commun. 2014;5(1):1–10. 10.1038/ncomms5022 PMC4090719 24959948

[39] Pio-Lopez L. Drug Repositioning Using Multiplex-Heterogeneous Network Embedding: A Case Study on SARS-CoV2. In Complex Networks & Their Applications X (R. M. Benito, C. Cherifi, H. Cherifi, E. Moro, L. M. Rocha, and M. Sales-Pardo, eds.), (Cham), pp. 731–741, Springer International Publishing, 2022.

[40] Blackiston D, Adams DS, Lemire JM, Lobikin M, Levin M. Transmembrane potential of glycl-expressing instructor cells induces a neoplastic-like conversion of melanocytes via a serotonergic pathway. Disease Models Mech. 2011;4(1):67–85. 10.1242/dmm.005561 PMC3008964 20959630

[41] Lobo D, Lobikin M, Levin M. Discovering novel phenotypes with automatically inferred dynamic models: a partial melanocyte conversion in xenopus. Sci Rep. 2017;7(1):41339. 28128301 10.1038/srep41339 PMC5269672

[42] Lobikin M, Lobo D, Blackiston DJ, Martyniuk CJ, Tkachenko E, Levin M. Serotonergic regulation of melanocyte conversion: A bioelectrically regulated network for stochastic all-or-none hyperpigmentation. Sci Signaling, **8**(397), 2015. 10.1126/scisignal.aac6609 26443706

[43] Buchta C, Kober M, Feinerer I, Hornik K. Spherical k-means clustering. J Statist Softw. 2012;50(10):1–22.

[44] Sive HL, Grainger RM, Harland RM. Early development of Xenopus laevis: a laboratory manual. New York: Cold Spring Harbor Laboratory Press; 2000.

[45] Nieuwkoop PD, Faber J. Normal table of Xenopus laevis (Daudin). A systematical and chronological survey of the development from the fertilized egg till the end of metamorphosis. Amsterdam: North-Holland Publishing Company, 1967.

[46] Varela I, Cadinanos J, Pendás AM, Gutiérrez-Fernández A, Folgueras AR, Sánchez LM, Zhou Z, Rodríguez FJ, Stewart CL, Vega JA, et al. Accelerated ageing in mice deficient in zmpste24 protease is linked to p53 signalling activation. Nature. 2005;437(7058):564–8. 16079796 10.1038/nature04019

[47] Worman HJ, Michaelis S. Prelamin a and zmpste24 in premature and physiological aging. Nucleus. 2023;14(1):2270345. 37885131 10.1080/19491034.2023.2270345 PMC10730219

[48] van Tintelen JP, Hofstra RM, Katerberg H, Rossenbacker T, Wiesfeld AC, du Marchie Sarvaas GJ, Wilde AA, van Langen IM, Nannenberg EA, van der Kooi AJ, et al. High yield of lmna mutations in patients with dilated cardiomyopathy and/or conduction disease referred to cardiogenetics outpatient clinics. Am Heart J. 2007;154(6):1130–9. 18035086 10.1016/j.ahj.2007.07.038

[49] Lo C-Y, Tjong Y-W, Ho JC-Y, Siu C-W, Cheung S-Y, Tang NL, Yu S, Tse H-F, Yao X. An upregulation in the expression of vanilloid transient potential channels 2 enhances hypotonicity-induced cytosolic ca2+ rise in human induced pluripotent stem cell model of hutchinson gillford progeria. PLoS One. 2014;9(1): e87273. 24475260 10.1371/journal.pone.0087273 PMC3903625

[50] Pio-Lopez L, Levin M. Aging as a loss of morphostatic information: a developmental bioelectricity perspective. Ageing Res Rev, p. 102310, 2024. 10.1016/j.arr.2024.102310 38636560

[51] Szenajch J, Wcislo G, Jeong J-Y, Szczylik C, Feldman L. The role of erythropoietin and its receptor in growth, survival and therapeutic response of human tumor cells: from clinic to bench-a critical review. Biochimica et Biophysica Acta (BBA)-Reviews on Cancer, **1806**(1), 82–95, 2010. 10.1016/j.bbcan.2010.04.002 20406667

[52] Roseti C, Cifelli P, Ruffolo G, Barbieri E, Guescini M, Esposito V, Di Gennaro G, Limatola C, Giovannelli A, Aronica E, et al. Erythropoietin increases gabaa currents in human cortex from tle patients. Neuroscience. 2020;439:153–62. 31047977 10.1016/j.neuroscience.2019.04.013

[53] Rose M, Kam P. Gabapentin: pharmacology and its use in pain management. Anaesthesia. 2002;57(5):451–62. 11966555 10.1046/j.0003-2409.2001.02399.x

[54] Costa B, Vale N. Understanding lamotrigine’s role in the cns and possible future evolution. Int J Mol Sci. 2023;24(7):6050. 37047022 10.3390/ijms24076050 PMC10093959

[55] Reid JG, Gitlin MJ, Altshuler LL. Lamotrigine in psychiatric disorders. J Clin Psychiatry. 2013;74(7):675–84. 23945444 10.4088/JCP.12r08046

[56] Liu M, Wei W, Stone CR, Zhang L, Tian G, Ding JN. Beneficial effects of trimetazidine on expression of serotonin and serotonin transporter in rats with myocardial infarction and depression. Neuropsychiatric Disease Treatment, pp. 787–797, 2018. 10.2147/NDT.S157441 PMC5859911 29588593

[57] Huang W, Cao L. Targeting gaba signalling for cancer treatment. Nat Cell Biology. 2022;24(2):131–2. 35145223 10.1038/s41556-021-00839-y

[58] Jayachandran P, Battaglin F, Strelez C, Lenz A, Algaze S, Soni S, Lo JH, Yang Y, Millstein J, Zhang W, et al. Breast cancer and neurotransmitters: emerging insights on mechanisms and therapeutic directions. Oncogene. 2023;42(9):627–37. 36650218 10.1038/s41388-022-02584-4 PMC9957733

[59] Huang D, Wang Y, Thompson JW, Yin T, Alexander PB, Qin D, Mudgal P, Wu H, Liang Y, Tan L, et al. Cancer-cell-derived gaba promotes -catenin-mediated tumour growth and immunosuppression. Nat Cell Biol. 2022;24(2):230–41. 35145222 10.1038/s41556-021-00820-9 PMC8852304

[60] Azuma H, Inamoto T, Sakamoto T, Kiyama S, Ubai T, Shinohara Y, Maemura K, Tsuji M, Segawa N, Masuda H, et al. -aminobutyric acid as a promoting factor of cancer metastasis; induction of matrix metalloproteinase production is potentially its underlying mechanism. Cancer Res. 2003;63(23):8090–6. 14678958

[61] El-Habr EA, Dubois LG, Burel-Vandenbos F, Bogeas A, Lipecka J, Turchi L, Lejeune F-X, Coehlo PLC, Yamaki T, Wittmann BM, et al. A driver role for gaba metabolism in controlling stem and proliferative cell state through ghb production in glioma. Acta Neuropathologica. 2017;133:645–60. 28032215 10.1007/s00401-016-1659-5 PMC5348560

[62] Bautista W, Perez-Alvarez V, Burczynski F, Raouf A, Klonisch T, Minuk G. Membrane potential differences and gabaa receptor expression in hepatic tumor and non-tumor stem cells. Canadian J Physiol Pharmacol. 2014;92(1):85–91. 10.1139/cjpp-2013-0226 24383877

[63] Schuller HM, Al-Wadei HA, Majidi M. Gamma-aminobutyric acid, a potential tumor suppressor for small airway-derived lung adenocarcinoma. Carcinogenesis. 2008;29(10):1979–85. 18310090 10.1093/carcin/bgn041 PMC2556972

[64] Ortega A. A new role for gaba: inhibition of tumor cell migration. Trends Pharmacol Sci. 2003;24(4):151–4. 12706998 10.1016/S0165-6147(03)00052-X

[65] Finnimore A, Roebuck M, Sajkov D, McEvoy R. The effects of the gaba agonist, baclofen, on sleep and breathing. Eur Respir J. 1995;8(2):230–4. 7758556 10.1183/09031936.95.08020230

[66] Noguchi H, Kitazumi K, Mori M, Shiba T. Binding and neuropharmacological profile of zaleplon, a novel nonbenzodiazepine sedative/hypnotic. Eur J Pharmacol. 2002;434(1–2):21–8. 11755161 10.1016/s0014-2999(01)01502-3

[67] Huddart R, Leeder JS, Altman RB, Klein TE. Pharmgkb summary: clobazam pathway, pharmacokinetics. Pharmacogenetics Genomics. 2018;28(4):110–5. 29517622 10.1097/FPC.0000000000000327 PMC5914180

[68] Bergmann KJ. Progabide: a new gaba-mimetic agent in clinical use. Clin Neuropharmacology. 1985;8(1):13–26. 2983890

[69] Sanna E, Busonero F, Talani G, Carta M, Massa F, Peis M, Maciocco E, Biggio G. Comparison of the effects of zaleplon, zolpidem, and triazolam at various gabaa receptor subtypes. Eur J Pharmacol. 2002;451(2):103–10. 12231378 10.1016/s0014-2999(02)02191-x

[70] Hardwick LJ, Philpott A. An oncologist’s friend: How xenopus contributes to cancer research. Dev Biol. 2015;408(2):180–7. 25704511 10.1016/j.ydbio.2015.02.003 PMC4684227

[71] Hardwick LJ, Philpott A. Xenopus models of cancer: expanding the oncologist’s toolbox. Front Physiol. 2018;9: 424568. 10.3389/fphys.2018.01660 PMC6277521 30538639

[72] Lobikin M, Chernet B, Lobo D, Levin M. Resting potential, oncogene-induced tumorigenesis, and metastasis: the bioelectric basis of cancer in vivo. Phys Biol. 2012;9(6): 065002. 23196890 10.1088/1478-3975/9/6/065002 PMC3528107

[73] Morokuma J, Blackiston D, Adams DS, Seebohm G, Trimmer B, Levin M. Modulation of potassium channel function confers a hyperproliferative invasive phenotype on embryonic stem cells. Proc Nat Acad Sci. 2008;105(43):16608–13. 18931301 10.1073/pnas.0808328105 PMC2575467

[74] Benkherouf AY, Taina K-R, Meera P, Aalto AJ, Li X-G, Soini SL, Wallner M, Uusi-Oukari M. Extrasynaptic -gabaa receptors are high-affinity muscimol receptors. J Neurochem. 2019;149(1):41–53. 30565258 10.1111/jnc.14646 PMC6438731

[75] Johnston GA. Muscimol as an ionotropic gaba receptor agonist. Neurochem Res. 2014;39:1942–7. 24473816 10.1007/s11064-014-1245-y

[76] Leach MJ, Wilson JA. Gaba receptor binding with 3h-muscimol in calf cerebellum. Eur J Pharmacol. 1978;48(3):329–30. 639861 10.1016/0014-2999(78)90092-4

[77] Scheel-Krüger J, Cools AR, Van Wel PM. Muscimol a gaba-agonist injected into the nucleus accumbens increases apomorphine stereotypy and decreases the motility. Life Sci. 1977;21(11):1697–702. 563960 10.1016/0024-3205(77)90250-8

[78] Shoulson I, Goldblatt D, Charlton M, Joynt RJ. Huntington’s disease: treatment with muscimol, a gaba-mimetic drug. Ann Neurol: Official J Am Neurol Assoc Child Neurol Soc. 1978;4(3):279–84. 10.1002/ana.410040316 152602

[79] Bartholini G. Pharmacology of the gabaergic system: effects of progabide, a gaba receptor agonist. Psychoneuroendocrinology. 1984;9(2):135–40. 6089242 10.1016/0306-4530(84)90032-5

[80] Wahab A, Heinemann U, Albus K. Effects of -aminobutyric acid (gaba) agonists and a gaba uptake inhibitor on pharmacoresistant seizure like events in organotypic hippocampal slice cultures. Epilepsy Res. 2009;86(2–3):113–23. 19535226 10.1016/j.eplepsyres.2009.05.008

[81] Maffini MV, Calabro JM, Soto AM, Sonnenschein C. Stromal regulation of neoplastic development: age-dependent normalization of neoplastic mammary cells by mammary stroma. Am J Pathol. 2005;167(5):1405–10. 16251424 10.1016/S0002-9440(10)61227-8 PMC1603788

[82] Kasemeier-Kulesa JC, Teddy JM, Postovit L-M, Seftor EA, Seftor RE, Hendrix MJ, Kulesa PM. Reprogramming multipotent tumor cells with the embryonic neural crest microenvironment. Dev Dyn. 2008;237(10):2657–66. 18629870 10.1002/dvdy.21613 PMC2570047

[83] Mintz B, Illmensee K. Normal genetically mosaic mice produced from malignant teratocarcinoma cells. Proc Nat Acad Sci. 1975;72(9):3585–9. 1059147 10.1073/pnas.72.9.3585 PMC433040

[84] Anderson B. Bioelectricity: a top-down control model to promote more effective aging interventions. Bioelectricity. 2024;6(1):2–12.

[85] Silver BB, Nelson CM. The bioelectric code: reprogramming cancer and aging from the interface of mechanical and chemical microenvironments. Front Cell Dev Biol. 2018;6:21. 29560350 10.3389/fcell.2018.00021 PMC5845671

[86] Zhao B-W, Su X-R, Yang Y, Li D-X, Li G-D, Hu P-W, Luo X, Hu L. A heterogeneous information network learning model with neighborhood-level structural representation for predicting lncrna-mirna interactions. Comput Struct Biotechnol J. 2024;23:2924–33. 39963422 10.1016/j.csbj.2024.06.032 PMC11832017

[87] Zhao B-W, Su X-R, Hu P-W, Ma Y-P, Zhou X, Hu L. A geometric deep learning framework for drug repositioning over heterogeneous information networks. Briefings Bioinf, **23,**(6), p. bbac384, 2022. 10.1093/bib/bbac384 36125202

[88] Zhao B-W, Su X-R, Yang Y, Li D-X, Li G-D, Hu P-W, You Z-H, Luo X, Hu L. Regulation-aware graph learning for drug repositioning over heterogeneous biological network. Inf Sci. 2025;686: 121360.

[89] Prouteau T, Dugué N, Guillot S. From communities to interpretable network and word embedding: an unified approach. J Complex Netw, **12**(6), p. cnae034, 2024.

[90] Zhang Y, Tiňo P, Leonardis A, Tang K. A survey on neural network interpretability. IEEE Trans Emerg Topics Computat Intell. 2021;5(5):726–42.

[91] Fan F-L, Xiong J, Li M, Wang G. On interpretability of artificial neural networks: a survey. IEEE Trans Radiation Plasma Med Sci. 2021;5(6):741–60. 10.1109/trpms.2021.3066428 PMC9105427 35573928

[92] Zhang X, Zhao Z, Li C, Zhang Y, Zhao J. An interpretable and scalable recommendation method based on network embedding. IEEE Access. 2019;7:9384–94.

[93] Hu W, Xu Y, Li Y, Li W, Chen Z, Tu Z. Bliva: A simple multimodal llm for better handling of text-rich visual questions. Proc AAAI Conf Artif Intell. 2024;38:2256–64.

[94] Lobo D, Malone TJ, Levin M. Towards a bioinformatics of patterning: a computational approach to understanding regulative morphogenesis. Biol Open. 2012;2(2):156–69. 23429669 10.1242/bio.20123400 PMC3575650

[95] Lobo D, Feldman EB, Shah M, Malone TJ, Levin M. A bioinformatics expert system linking functional data to anatomical outcomes in limb regeneration. Regeneration. 2014;1(2):37–56. 25729585 10.1002/reg2.13 PMC4339036

